# Influence of annealing pretreatment in different atmospheres on crystallization quality and UV photosensitivity of gallium oxide films

**DOI:** 10.1039/d3ra07568k

**Published:** 2024-02-02

**Authors:** Wen-Jie Chen, Hong-Ping Ma, Lin Gu, Yi Shen, Ruo-Yun Yang, Xi-Yuan Cao, Mingyang Yang, Qing-Chun Zhang

**Affiliations:** a Institute of Wide Bandgap Semiconductors and Future Lighting, Academy for Engineering & Technology, Fudan University Shanghai 200433 China hpma@fudan.edu.cn; b Shanghai Research Center for Silicon Carbide Power Devices Engineering & Technology, Fudan University Shanghai 200433 China; c Institute of Wide Bandgap Semiconductor Materials and Devices, Research Institute of Fudan University in Ningbo Zhejiang 315327 China; d Key Laboratory of Instrumentation Science & Dynamic Measurement, School of Instrument and Electronics, North University of China Taiyuan 030051 China; e Key Laboratory of Marine Materials and Related Technologies, Zhejiang Key Laboratory of Marine Materials and Protective Technologies, Ningbo Institute of Materials Technology and Engineering, Chinese Academy of Sciences Ningbo 315201 China

## Abstract

Due to their high wavelength selectivity and strong anti-interference capability, solar-blind UV photodetectors hold broad and important application prospects in fields like flame detection, missile warnings, and secure communication. Research on solar-blind UV detectors for amorphous Ga_2_O_3_ is still in its early stages. The presence of intrinsic defects related to oxygen vacancies significantly affects the photodetection performance of amorphous Ga_2_O_3_ materials. This paper focuses on growing high quality amorphous Ga_2_O_3_ films on silicon substrates through atomic layer deposition. The study investigates the impact of annealing atmospheres on Ga_2_O_3_ films and designs a blind UV detector for Ga_2_O_3_. Characterization techniques including atomic force microscopy (AFM), X-ray diffraction (XRD) and X-ray photoelectron spectroscopy (XPS) are used for Ga_2_O_3_ film analysis. Ga_2_O_3_ films exhibit a clear transition from amorphous to polycrystalline after annealing, accompanied by a decrease in oxygen vacancy concentration from 21.26% to 6.54%. As a result, the response time of the annealed detector reduces from 9.32 s to 0.47 s at an external bias of 10 V. This work demonstrates that an appropriate annealing process can yield high-quality Ga_2_O_3_ films, and holds potential for advancing high-performance solar blind photodetector (SBPD) development.

## Abstract 2

Due to their high wavelength selectivity and strong anti-interference capability, solar-blind UV photodetectors hold broad and important application prospects in fields like flame detection, missile warnings, and secure communication.

## Graphical abstract

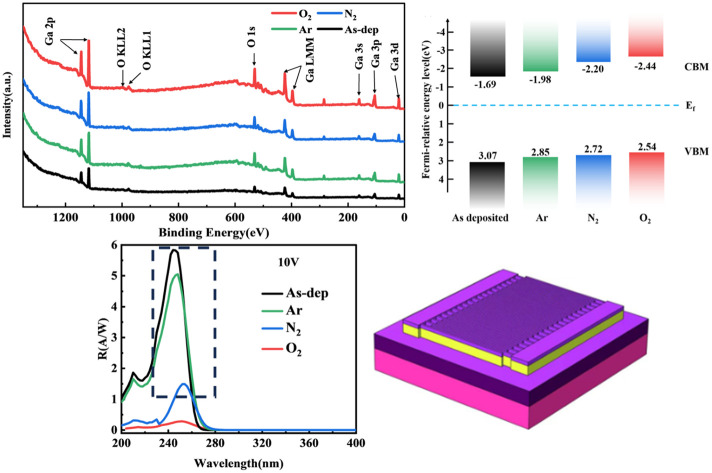

## 1. Introduction

The earth's atmosphere strongly absorbs and scatters ultraviolet light, primarily due to ozone. As a result, sunlight with wavelengths ranging from 200 to 280 nm has limited penetration of the earth's atmosphere. This specific range of light is commonly referred to as ‘solar-blind’ or the ‘solar-blind band’ due to its restricted reach.^1,2^ The intensity of radiation in this solar blind region is significantly lower than that in the visible region. Combined with the low natural background, photodetectors designed to operate within this spectral band offer several advantages, including a high signal-to-noise ratio, excellent sensitivity, and a relatively low false alarm rate.^3,4^

At present, commonly used wide-band gap semiconductor materials for solar-blind UV photodetectors primarily consist of Ga_2_O_3_, ZnMgO, and AlGaN.^5–7^ The growth of AlGaN and ZnMgO involves extremely high-temperature requirements and a complex epitaxy process, contributing to a relatively higher cost of film preparation. In addition, due to the problems of alloy material preparation technology itself, for example, MgZnO with high Mg element ratio is not able to prepare reliable and stable wurtzite structure to meet the requirements of device preparation and light signal detection. The stability of the semiconductor film is compromised, thereby negatively impacting the feasibility of large-scale film growth. In comparison to other wide-gap semiconductor materials, Ga_2_O_3_ possesses a relatively suitable bandgap width (4.5–4.9 eV),^8^ making it well-suited for solar-blind UV detection without the need for additional complex alloying processes. Additionally, its absorption coefficient near the absorption edge reaches 10^5^ cm^−1^, establishing it as a natural material for solar-blind UV detection with promising practical utility.^9^

β-Ga_2_O_3_ continues to serve as the primary structure for gallium oxide-based materials and their solar-blind ultraviolet detectors. However, producing high-quality β-Ga_2_O_3_ films typically demands elevated growth temperatures (>600 °C). The preparation process is complex, and equipment costs are high.^10^ As flexible optoelectronic devices and large-area photovoltaic devices advance, the demand grows for materials with low cost, simplified preparation processes, scalability in production and low preparation temperature. Consequently, researchers are increasingly focusing on the low-temperature growth of amorphous materials.^11,12^ In recent years, a series of reports have emerged on solar-blind UV photodetectors based on amorphous Ga_2_O_3_, and some devices have exhibited excellent responsiveness characteristics. However, due to the simultaneous presence of a substantial number of oxygen vacancy defects and grain boundaries within amorphous Ga_2_O_3_ materials, a comprehensive understanding of the mechanisms through which they influence the physical properties of the materials and the performance of optoelectronic devices remains elusive. This lack of clarity significantly constrains the optimal design of both materials and devices.

Recently, an increasing number of scientists have initiated investigations into the growth patterns of amorphous Ga_2_O_3_ films and their corresponding photoelectric properties. Qian *et al.* synthesized both β-Ga_2_O_3_ and amorphous films using Molecular Beam Epitaxy (MBE).^13^ XPS analysis reveals that the surface of the magnetron-sputtered film exhibits roughness, suggesting a potential production of additional surface defect states during the sputtering process. The concentration of oxygen vacancies in the film was measured, revealing that the oxygen vacancies in the sputtering-deposited film were twice as numerous as those generated by MBE.

In 2014, Guo *et al.*^14^ examined the effects of different growth temperatures on the structure, surface, and optical properties of Ga_2_O_3_ films using metal–organic deposition on a C-plane sapphire substrate. As the temperature increased, the crystallization properties, grain size, and surface roughness of the Ga_2_O_3_ films also increased. In 2017, Cui *et al.*^15^ from the Institute of Physics, Chinese Academy of Sciences, employed magnetron sputtering to grow Ga_2_O_3_ amorphous films on both quartz and flexible substrates at room temperature. Rafique *et al.*^16^ grew β-Ga_2_O_3_ films on a C-plane sapphire substrate utilized low-pressure chemical vapor deposition.

In 2020, a Korean team used HVPE method to grow α-Ga_2_O_3_ films with good crystallinity and smooth surface morphology on sapphire substrate.^17^ The development of the thin film will promote the further development of α-Ga_2_O_3_ in the field of optoelectronic devices. In 2021, Wang *et al.*^18^ used PLD system to deposit Ga_2_O_3_ film on sapphire substrate, and raised the growth temperature from room temperature to 600 °C. They found that the deposition rate of the film decreased with the increase of temperature, and the existence of non-lattice oxygen vacancy was confirmed. High quality single crystal Ga_2_O_3_ films with high visible and near-infrared transmittance, large grain size and smooth surface were obtained. In 2023, X. Ji.^19^*et al.*, from Shandong University, prepared amorphous Ga_2_O_3_ films by RF magnetron sputtering technology, and reported a Schottky photodiode with asymmetric electrode. Under a bias voltage of 5 V, the *I*_dark_ is as low as 6.6 pA, and the light–dark current ratio is as high as 2.3 × 10^6^. Responsiveness up to 1021.8 A W^−1^; this result is comparable to or better than the reported high performance β-Ga_2_O_3_ Schottky photodiodes and provides a feasible way to achieve large area, low cost, high contrast and high detection sensitivity of solar blind imaging.

In contrast to methods such as MOCVD, ALD enables the growth of thin films at low temperatures, for example, below 200 °C. This feature is valuable for preparing materials on flexible substrates. Moreover, ALD is considered the preferred method for precisely controlling the thickness of micro or nanoscale structures, even at the atomic level, and for conformal covering on substrates with low impurity content and pinhole density. Consequently, when combined with appropriate annealing treatment, the crystal quality of Ga_2_O_3_ thin film materials can be further enhanced.

In this work, 80 nm Ga_2_O_3_ films were grown using the atomic layer deposition (ALD) technique. Subsequently, the films were annealed for 30 minutes at 800 °C under O_2_, N_2_, and Ar. The surface morphology and chemical composition of each film were characterized to analyze the effects of different annealing atmospheres on oxygen vacancy concentration and film quality. Additionally, a Ga_2_O_3_ metal–semiconductor–metal (MSM) SBPD was fabricated based on the thin film. The photodetection properties of the SBPD were analyzed, and the photo-response mechanism of the Ga_2_O_3_ thin films under various annealing conditions was investigated.

## 2. Experimental section

### 2.1 Preparation of Ga_2_O_3_ film

Ga_2_O_3_ films were grown on a Si substrate using the BENEQ TFS200 ALD system (BENEQ, Finland). Prior to deposition, the substrate underwent cleaning, followed by rinsing with deionized water and drying within an N_2_ atmosphere. During deposition, the precursors employed were trimethylgallium (TMG) for Ga and O_2_ plasma for O. TMG was stored in stainless steel bottles at 10 °C. Activation of the O_2_ plasma was performed at 200 W. All film depositions took place at a substrate temperature of 200 °C, under a pressure of ∼3.5 mbar. The thickness of Ga_2_O_3_ films grown in this study is 80 nm.

### 2.2 Sample characterization

The surface morphology of a randomly chosen 500 nm × 500 nm area was examined utilizing AFM (Bruker, Icon) through a non-contact method, in order to characterize the microstructure and morphology of the films. The sample is tested based on synchrotron grazing-incidence X-ray diffraction (GIXRD) measurements, using X-rays with A wavelength of 0.27 A and a scanning range of 20°–90°. The chemical bonds of the films were characterized through XPS (SPECS, Berlin, Germany) using a monochromatic aluminum source (*hν* = 1486.6 eV). A narrow scan resolution of 0.1 eV was adopted. All XPS spectra were calibrated using a combined energy of 284.6 eV for C 1s peaks. Photoluminescence performance was assessed with a steady state/transient fluorescence spectrometer (FLS980) in the photoemission test section, subjecting each sample to illuminations at wavelengths of 200–500 nm with 1 nm step-size. The analytical process also encompassed Raman analysis (LabRam HR Evolution; Thermo Fischer DXR).

To examine the *I*–*V* characteristics and time response of the detector under both dark and light conditions, we employed the spectral response measuring equipment, model Thermo IS10, in this study. This equipment included a light source, a monochromator, a measurement chamber insulated from external light, a phase-locked amplifier, a data measurement source table, and a control computer. Additionally, a 254 nm mercury lamp light source (equipped with a timing control module), a probe station for sample handling, and an Agilent 4155B test instrument for device performance testing and analysis were part of the experimental setup. The timing module controlled the on–off cycles of UV radiation. The Agilent 4155B test instrument was used to apply bias to the device sample on the probe table, facilitating the collection and recording of the device's output current.

## 3. Results and discussion

### 3.1 Morphology and microstructure

In ALD-prepared amorphous Ga_2_O_3_, a significant issue persists: the presence of defects, particularly oxygen vacancies. Crystalline oxide semiconductors have lower oxygen vacancy levels compared to amorphous oxide semiconductors, which exhibit higher oxygen vacancy contents.^20^ Oxygen vacancy defects generally scatter carriers, reducing their mobility and significantly affecting the material's mechanical properties and thermal conductivity. Annealing holds significant importance as a technology in the semiconductor industry. High-temperature annealing supplies sufficient energy for gallium and oxygen atoms to transition to their appropriate lattice positions, allowing them to reconfigure into the crystal phase.^21,22^ This promotes the selective growth of the film, subsequently facilitating the effective release of stress between the film and substrate and eliminating dislocation defects. However, there is limited research on the influence of annealing on oxygen vacancy defects in α-Ga_2_O_3_ films and their performance in solar-blind UV detection. In this study's annealing experiment design, three sample groups underwent 30 minutes of constant-temperature annealing in argon, nitrogen, and oxygen atmospheres. The outcomes were then compared with a set of data from unannealed samples.

**Fig. 1:**
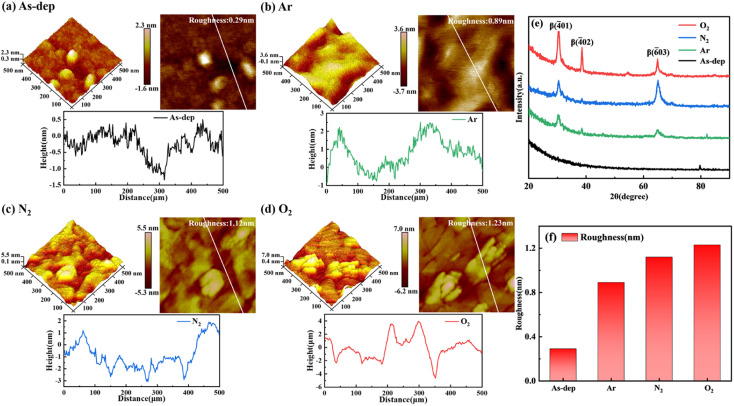
(a)–(d) Two- and three-dimensional morphology and roughness of Ga_2_O_3_ thin films under varying annealing atmospheres: (a) unannealed, (b) Ar-annealed, (c) N_2_-annealed, (d) O_2_-annealed. (e) Grazing incident XRD patterns of Ga_2_O_3_ films prepared using different annealing atmospheres. (f) Surface roughness of Ga_2_O_3_ films prepared under diverse annealing atmospheres.

To analyze the influence of distinct annealing atmospheres on the surface morphology of β-Ga_2_O_3_ films, we characterized the films obtained under various annealing atmospheres using AFM. Fig. 1(a)–(d) illustrates the AFM 2D and 3D morphology, as well as the results of surface grain analysis, for Ga_2_O_3_ films annealed in Ar, N_2_, and O_2_ atmospheres, respectively. The AFM images were acquired within a scanning area of 500 nm × 500 nm. The 3D AFM representation clearly depicts that the grains in the unannealed sample are relatively small and disordered, whereas the annealed film surface generally exhibits larger grains.^23,24^

After annealing, the sample's grain height increases from 2.3 nm to 7 nm, likely due to the transition from an amorphous to a crystalline phase. Fig. 1(e) illustrates the grazing incidence XRD pattern of β-Ga_2_O_3_ films annealed under varying nitrogen atmosphere temperatures. These XRD patterns reveal no discernible diffraction peaks in the unannealed film, indicating its amorphous state. However, in the annealed samples, distinct diffraction peaks corresponding to β (−401), β (−402), and β (−603) crystallographic planes appear at around 2*θ* = 26°, 39°, and 67°, respectively.^25,26^ Moreover, these peaks progressively intensify in samples annealed under different atmospheres—specifically Ar, N_2_, and O_2_.

From this, it becomes evident that annealing facilitates the provision of energy required for the rearrangement of Ga and O atoms. This, in turn, expedites the migration of atoms within the film to their appropriate lattice positions, promoting a closer alignment of the film with the β phase. The process of atomic rearrangement also helps mitigate the internal stresses resulting from lattice mismatch or distortion during the growth of amorphous Ga_2_O_3_, thereby enhancing the film's quality to a certain extent. Different atmospheres yield different effects on the results. For instance, annealing in an O_2_ atmosphere introduces O atoms into the film's interior, accelerating the transition to the β phase and intensifying the peak value. These observations correspond to AFM images.

The 2D AFM image primarily analyzes the RMS roughness of the film. The RMS roughness of the unannealed films measures 0.29 nm, whereas for the films annealed in Ar, N_2_, and O_2_ atmospheres, the RMS roughness values are 0.89 nm, 1.12 nm and 1.23 nm., respectively. As depicted in Fig. 1(f), the surface RMS roughness of Ga_2_O_3_ films increases post the annealing process. This phenomenon can be attributed to the reduction in surface energy within the film due to annealing, which in turn promotes the polymerization and growth of small grains. Conversely, annealing provides sufficient energy that aids grain polymerization, resulting in increased grain size and corresponding roughness.^27^

### 3.2 Composition analysis

**Fig. 2:**
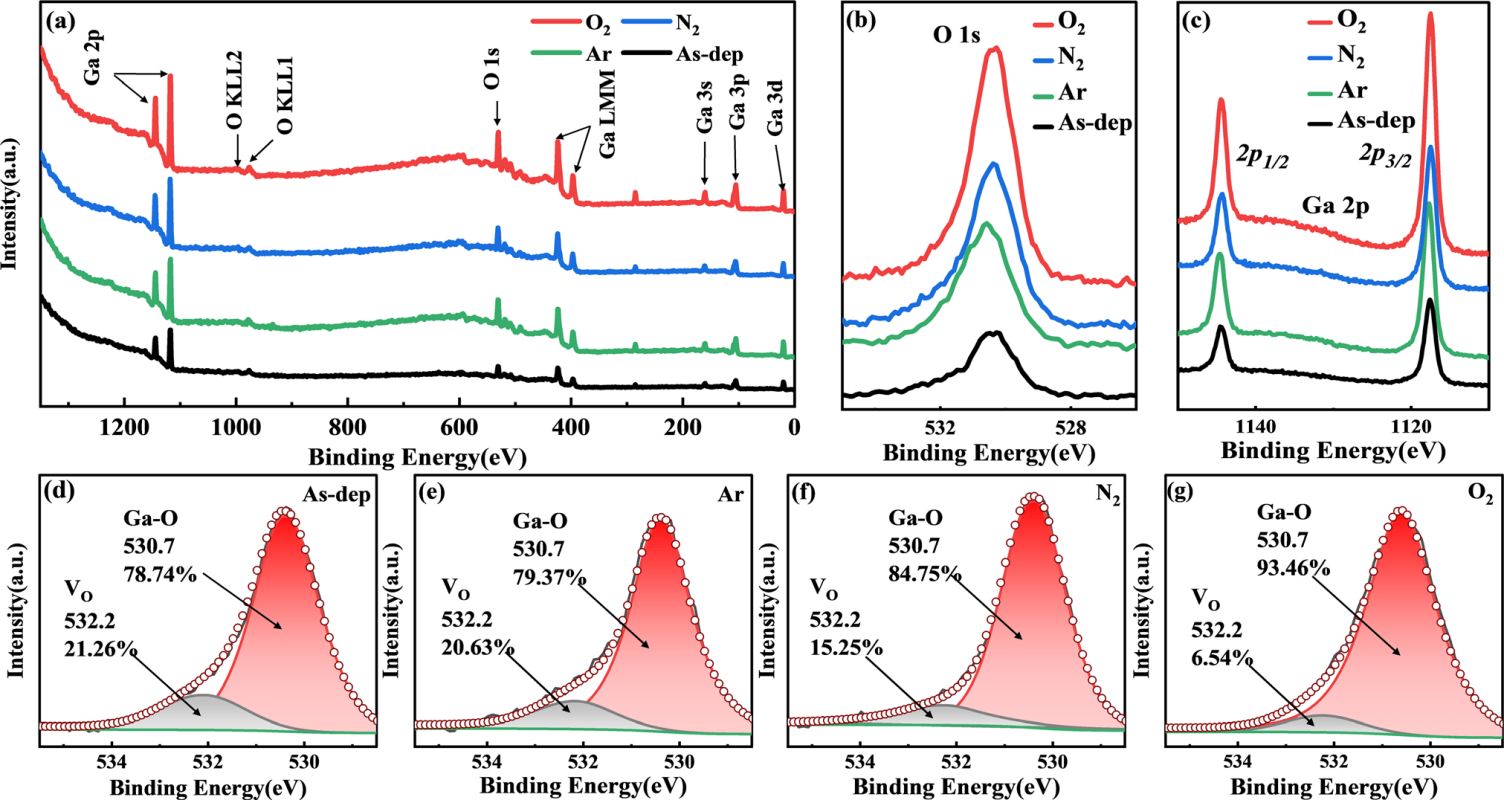
XPS analyses of Ga_2_O_3_ samples annealed under different atmospheres: (a) survey peaks; (b) O 1s spectra; and (c) Ga 2p spectra. The O 1s spectra of Ga_2_O_3_ films annealed in various atmospheres are shown for (d) unannealed, (e) Ar, (f) N_2_, and (g) O_2_.

To further analyze the concentration, microstructure, chemical valence, and composition of elements in the film, XPS was employed to measure the chemical bonds of elements within the Ga_2_O_3_ film. The calibration process relied on the C 1s emission line. The complete XPS scan spectrum is presented in Fig. 2(a), which annotates and identifies peaks corresponding to all elements. Notably, detailed analysis was carried out on the O 1s and Ga 2p peaks, as depicted in Fig. 2(b) and (c). Following annealing, changes in Ga–O bond concentration and the redistribution of charge around constituent atoms led to variations in peak intensity. This trend exhibited a progressive increase in intensity from the non-annealed state to treatments involving Ar, N_2_, and O_2_.

To analyze the impact of oxygen vacancies on photoelectric properties, we examined the O 1s peaks of each film. As depicted in Fig. 2(d)–(g), the Ga_2_O_3_ sample displays two subpeaks centered at 530.7 and 532.2 eV. The peak at 530.7 eV corresponds to lattice oxygen within the Ga_2_O_3_ film,^28,29^ while the peak at 532.2 eV is linked to O_2_ ions situated in the oxygen vacancy within the GaO_*X*_ matrix, commonly known as a Vo-like bond.^30^ In the unannealed Ga_2_O_3_ film, the ratio of oxygen vacancies to lattice oxygen is 0.27 : 1. However, in the annealed Ga_2_O_3_ film, the oxygen vacancy defect is notably reduced, with the ratio of oxygen vacancies to lattice oxygen in the Ar, N_2_, and O_2_ annealed films being 0.26 : 1, 0.18 : 1, and 0.07 : 1, respectively.

The alteration in the intensity ratio of the two sub-peaks associated with the Ga–O bond indicates a decrease in the concentration of oxygen vacancies following the annealing treatment. Different atmospheres influence the extent of reduction in oxygen vacancies. In an Ar atmosphere, the inert gas maintains relative stability, causing the impact of film annealing to closely resemble that of the untreated film. During N_2_ atmosphere annealing, a fraction of N atoms infiltrates the film to occupy vacant oxygen sites.^31^ Furthermore, N atoms within the lattice capture available O atoms to a certain extent, thus facilitating rearrangement crystallization. Annealing in an O_2_ atmosphere results in a significant influx of O atoms into the film, effectively replenishing the oxygen vacancies and markedly reducing their presence.^32^

### 3.3 Optical testing and photoemission analysis

**Fig. 3:**
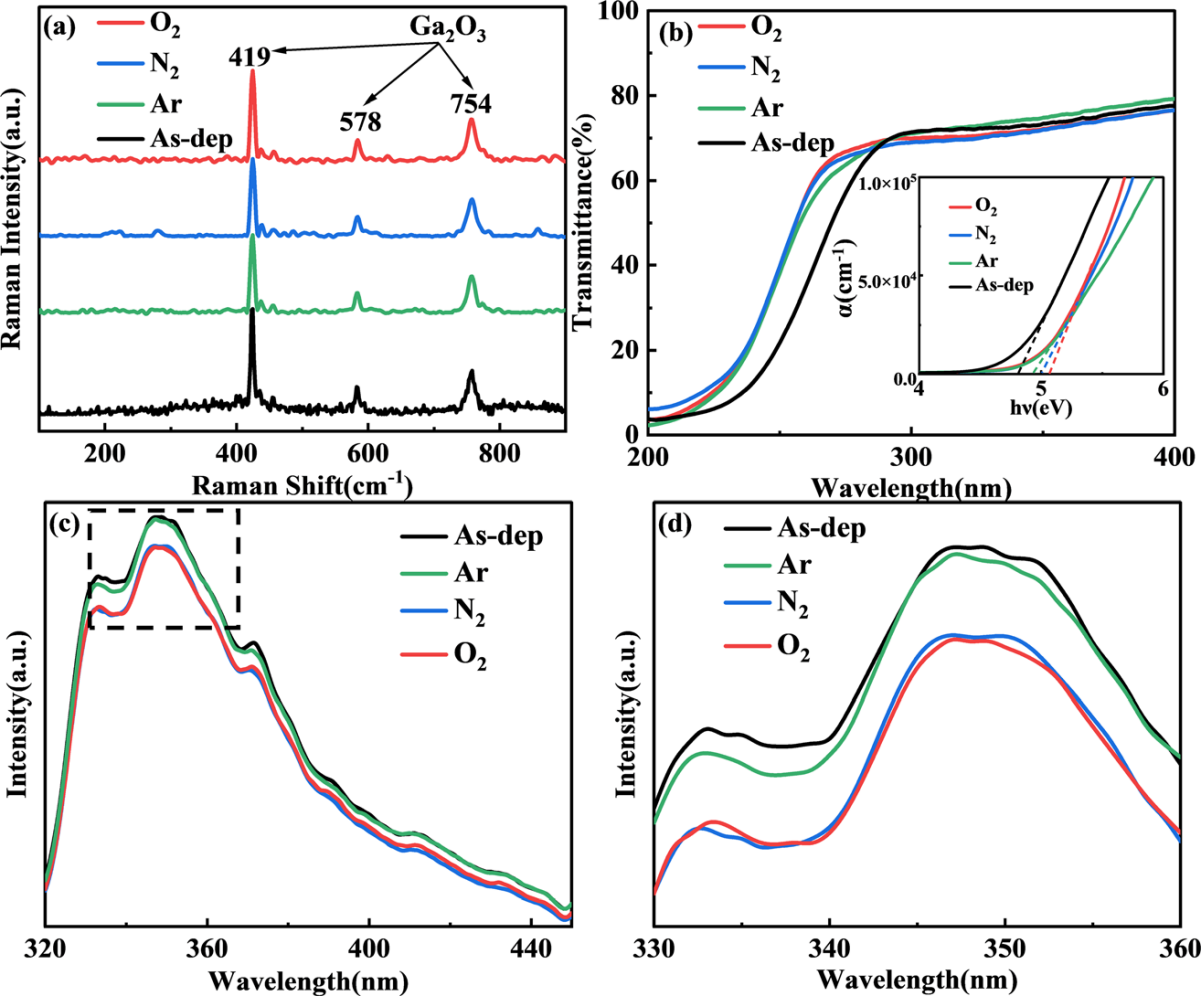
(a) Raman spectra of Ga_2_O_3_ annealed under different atmospheric conditions. (b) Ultraviolet light transmittance and optical band gap of Ga_2_O_3_ annealed in different atmospheres. (c) PL spectra of Ga_2_O_3_ films under various annealing conditions. (d) Magnified PL spectral image within the wavelength range of 330–360 nm.

In order to prepare a high-performance solar-blind UV detector based on the already prepared film, it is necessary to understand the optical properties of the material itself in advance. Fig. 3(a) illustrates the Raman spectra of Ga_2_O_3_ subjected to annealing in different atmospheres. β-Ga_2_O_3_ has a monoclinic crystal structure, belonging to the C 2*h* space group. As a result, the cells of β-Ga_2_O_3_ comprise Ga_2_O_6_ octahedrons and GaO_4_ tetrahedrons.^33^ Raman activity patterns can be categorized as follows: high-frequency peaks surpassing 600 cm^−1^ pertain to the stretching and bending of the GaO_4_ tetrahedron; mid-frequency peaks between 310 and 480 cm^−1^ correspond to the deformation of the Ga_2_O_6_ octahedron; and low-frequency peaks under 200 cm^−1^ are related to the translation of the tetrahedral-octahedral chain.^34,35^ Therefore, the standard peaks for β-Ga_2_O_3_ are typically characterized by high-frequency peaks above 600 cm^−1^ and low-frequency peaks below 200 cm^−1^.

The peaks within the 400–600 cm^−1^ range represent the standard peaks of GaO_6_, and those within the 200–400 cm^−1^ range mostly correspond to the standard peaks of amorphous Ga_2_O_3_. In the 200–400 cm^−1^ range, the spectral noise fluctuations of annealed Ga_2_O_3_ films are significantly smaller than those of the unannealed samples. This suggests that the annealed samples have transitioned from their amorphous state, resulting in reduced residual strain and a lower dislocation density. Moreover, there is a successive enhancement in the peaks corresponding to Ar, N_2_, and O_2_ within the annealed Ga_2_O_3_ films. This enhancement indicates a gradual shift towards a more distinct single β phase structure. This observation is consistent with earlier findings from AFM and graze-incidence XRD analyses.

Fig. 3(b) depicts the computation results for ultraviolet transmittance and optical band gap. Due to the thickness of the sample, the transmittance of UVA and UVB wavelengths above 280 nm remains only slightly higher than 60%. However, for the UVC band ranging from 200 nm to 280 nm, all films consistently maintain a relatively high level of transmittance. A certain degree of redshift is evident in the annealed film when compared with the unannealed film. Using the transmittance spectrum, the absorption coefficient is determined through the following calculation.

UV/visible light photometers operate based on the principle of the law of light absorption, as described by the formula:^36^

1 *I* = *I*_0_e^−*αd*^

where *α* represents the absorption coefficient, *I*_0_ refers to the steady-state photocurrent, and *d* is the film thickness. The calculation method of the absorption coefficient is as follows:^33^2
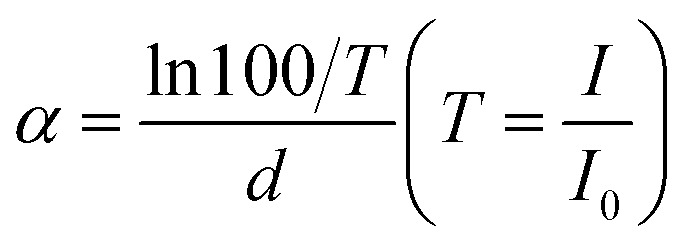


As Ga_2_O_3_ is a direct band gap semiconductor, its optical band gap and light absorption adhere to the following expression:^36^

3 (*ah*ν)^2^ = *B*(*hν* − *E*_g_)

where *h* is the Planck constant, *ν* is the incident light frequency, *B* is a constant, and *E*_g_ stands for the optical band gap. The fitting results are displayed in Fig. 3(b). The value of the optical band gap, *E*_g_, is obtained by extending the intercept of the linear segment of the graph along the horizontal axis of *hν*. Based on the calculated outcomes, the optical band gap value of the unannealed film measures 4.78 eV. Additionally, the optical band gap values for the film annealed in Ar, N_2_, and O_2_ atmospheres are 4.90 eV, 4.99 eV, and 5.06 eV, respectively.^37,38^ This demonstrates that annealing induces a band shift in Ga_2_O_3_, resulting in an increased band gap, a phenomenon that is also influenced by the annealing atmosphere.

Fig. 3(d) illustrates the PL spectra of samples subjected to different annealing conditions. A 240 nm excitation light wavelength was employed for these measurements. In the 320–450 nm range, all spectral peaks exhibit consistent linear shapes, with their emission peak positions remaining independent of the sample's morphology. The observable luminescence peak at 350 nm is a result of laser diffraction. Variations in annealing conditions lead to slight differences in the intensity of each spectral peak. When the Ga_2_O_3_ material is annealed in argon, the oxygen vacancy defect of the film does not decrease significantly, resulting in the emission peak value similar to that of the unannealed film. However, oxygen atoms in Ga_2_O_3_ annealed in a nitrogen atmosphere escape from the sample, resulting in a large number of oxygen vacancy defect donors.^39^ However, due to the tunneling effect, electrons in the donor are captured by either gallium (Ga) or gallium–oxygen (Ga–O) vacancies, forming trapping excitons and producing emission. The lowest peak value was obtained by annealing in an oxygen atmosphere, which confirmed the lowest oxygen vacancy defect concentration in the film.

In this paper, the 350 nm excitation light is attributed to vacancy-type defects (V_O_, V_Ga_–V_O_). Vacancy defects can be categorized into two types: oxygen vacancies (V_O_) acting as donor defects, and gallium–oxygen vacancy pairs (V_Ga_–V_O_) acting as acceptor defects.^39,40^ Both types result in the complex emission of defects. The excitation light near 350 nm is more common for some β-Ga_2_O_3_ nanomaterials for blue-purple luminescence bands. Experimental findings indicate that annealing leads to a reduction in defect concentration and a decline in the peak value of the excitation light. The lowest peak value achieved through annealing in an oxygen atmosphere confirms the film's lowest concentration of oxygen vacancy defects, aligning with the outcomes observed in the O 1s peak of XPS analysis.^41^

### 3.4 Photodetector test

Given the challenges associated with producing high-quality P-type Ga_2_O_3_ films, the PN junction detectors formed using β-Ga_2_O_3_ and other P-type materials consistently exhibit lattice mismatches. This, in turn, leads to heterogeneous epitaxy in the film, consequently compromising the film's quality. Moreover, the intricate preparation process required for achieving high-quality films exacerbates this concern, substantially limiting the practical advancement of this technology. In contrast, the MSM structure-based detector has gained significant attention due to its straightforward fabrication process, minimal junction capacitance, and negligible leakage. To investigate how annealed pairs influence the photoelectric properties of α-Ga_2_O_3_, we produced metal–semiconductor–metal (MSM) photodetectors using these sample films. The device features an interdigital electrode with dimensions: a 20 μm width, 20 μm interdigital spacing, and 500 μm length. The configuration comprises 25 pairs of interdigital electrodes, with each Ti/Au electrode measuring approximately 20/50 nm in thickness.

**Fig. 4:**
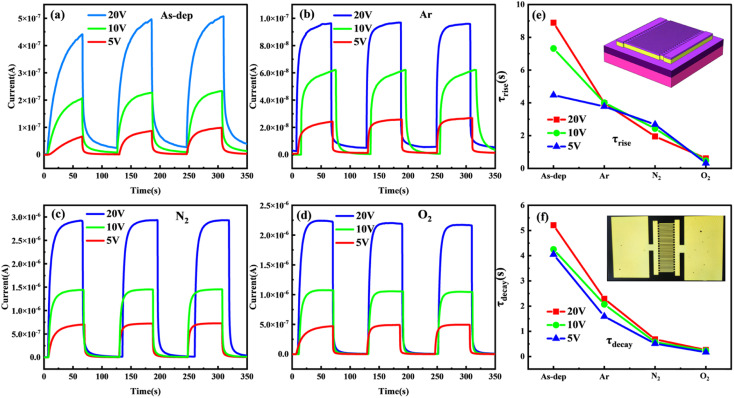
Dynamic IV-T results at different bias voltages: (a) unannealed, (b) Ar, (c) N_2_, and (d) O_2_. The (e) *τ* increases and (f) *τ* decays for Ga_2_O_3_ detectors in different annealing atmospheres (the illustrations in (e) and (f) are the schematic diagram of the detector structure and the image of the interdigital electrode, respectively).

To investigate the impact of annealing conditions on the photoelectric properties of ALD-grown Ga_2_O_3_ films, we prepared the corresponding MSM-SBPD as shown in Fig. 4(e) and (f). Key quality metrics, such as the light-to-dark current ratio (PDCR), response rate (*R*), and normalized detectivity (D*),^39,40^ were used to evaluate the performance of the Ga_2_O_3_ photodetector. The photocurrent denotes the device's output current when a bias voltage is applied across its positive and negative electrodes, while it is subjected to a specific wavelength of radiation. Due to the reduction in semiconductor resistivity driven by photoexcitation, the photocurrent is generally significantly higher than the dark current.

Photogenerated current is defined as the difference between photocurrent and dark current, and its magnitude reflects, to a certain extent, the semiconductor materials' photoelectric conversion ability. The formula used for calculating PDCR is as follows:^42^

4 PDCR = (*I*_light_ − *I*_dark_)/*I*_dark_

where *I*_dark_ is dark current and *I*_light_ is photocurrent.

The optical responsivity signifies the photoelectric conversion capability of the photoelectric conversion device concerning a given optical signal. When placed under a specific bias voltage, the optical responsivity corresponds to the ratio of the photogenerated current to the incident light power. The magnitude of the optical responsivity indicates the strength of the device's photoelectric conversion ability. The calculation formula is as follows:^43^

5 *R* = (*I*_light_ − *I*_dark_)/*P*

where *P* represents the optical power of the applied light. Generally speaking, the development of light detectors will pursue the increase of these two performance parameters.

The signal-to-noise ratio refers to the ratio of signal to noise that is converted into the current output value during detector detection. This parameter holds significant importance for the detector's detection ability. However, noise generation is a complex process encompassing internal thermal noise of the device, shot noise resulting from material excitation by light, flicker noise associated with surface traps, back-bottom noise from the external environment, and other factors. Consequently, the signal-to-noise ratio for a 1 W power light incident on a 1 cm^2^ device area defines the normalized detectivity (D*).^44^ This term essentially quantifies the detector's capability to detect the minimum optical signal during normal operation, the calculation is as follows:^40^6
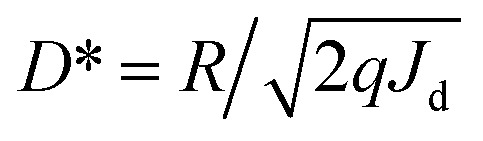
where *R* represents the optical response intensity of the device, *q* signifies the amount of electron charge. *J*_d_ corresponds to the dark current density of the device, which can be calculated by dividing the size of the dark current by the effective area. Evidently, in the context of optical detection, a higher detectivity D* indicates superior performance.^45^

Repeatability is also an important factor for determining the long-term stable dynamic operation of the photodetector. To test the repeatability of the amorphous Ga_2_O_3_ ultraviolet detector, we evaluated the time-dependent optical response of the device at a wavelength of 254 nm. During the measurement, a 254 nm light source, with a power of 90 μW cm^−2^, was turned on and off every 60 seconds.^46^ The figure illustrates that all photodetectors exhibit excellent repeatability and stability during operation.

Further analysis of the response time for each detector reveals a higher photocurrent for the same device under a higher bias voltage. Additionally, the response time varies when the film is switched on and off in different annealing atmospheres. The response time (*τ*) comprises the rise time (*τ*_rise_) and the decay time (*τ*_decay_). *τ*_rise_ is defined as the time taken for *I*_ph_ to increase from 10% of the steady state value to 90% of the maximum value, while *τ*_decay_ is defined as the time taken for *I*_ph_ to decrease from 90% to 10%.^47,48^ The test curve is fitted using a double exponential relationship equation with the expression from ref. 43:

7 *I* = *I*_0_ + *A*e^−*t*/*τ*_1_^ + *B*e^−*t*/*τ*_2_^

where *A* and *B* are constants, *t* stands for time, and *τ*_1_ and *τ*_2_ are relaxation time constants.

The fitting outcomes are shown in Fig. 4(e) and (f). Generally, the rise time (*τ*_rise_) for each device tends to exceed the decay time (*τ*_decay_). This could be attributed to the device's interaction with 254 nm light under applied voltage. The increased generation of electron–hole pairs during this interaction does not lead to an instantaneous change in current with external conditions. This delay is due to the required migration time for carriers. Notably, the photocurrent demonstrates enhancement with higher applied voltage. For instance, the application of a higher bias voltage of 20 V induces greater electron excitation and mobility. This effect accelerates the absorption of photogenerated carrier separation by the electrode, consequently expediting the rise time.

The annealing duration of the film varies across different atmospheres. The sequence from fastest to slowest annealing is observed in the following order: film annealed in O_2_, N_2_, and Ar atmospheres, followed by the non-annealed film. The non-annealed film demonstrates the longest response time, with a rise time of 9.32 seconds under a 10 V bias. Conversely, the film annealed in an oxygen atmosphere shows the shortest response time, displaying alterations in *τ* with an increase of 0.45 seconds and a decrease of 0.17 seconds at a 5 V bias.

In a semiconductor material, defects may give rise to two types of carrier-effect centers: trap centers and recombination centers. Trap centers can capture a single type of carrier (either an electron or a hole). Typically found at shallow energy levels, they capture electrons during the transition of excited valence band electrons, thereby increasing the device's *τ* (time constant). Additionally, they can also trap electrons descending from the conduction band after the excitation is removed, further extending the device's *τ* attenuation. Recombination centers, operating through a different mechanism, generally exist at deep energy levels. They facilitate the recombination of non-equilibrium carriers, thereby reducing the device's recovery time.^49–52^

Combined with the prior analysis of film quality, Fig. 4 illustrates that distinct atmospheres exert a more pronounced influence on the increased *τ* of the detector subsequent to annealing the device substrate. Hence, the subsequent improvement in epitaxial quality of gallium oxide films primarily signifies a noteworthy reduction in material defects functioning as trap centers, while the density of composite centers experiences marginal change. Moreover, imperfections like oxygen vacancies and gallium vacancies commonly manifest as trap centers at deep levels within the material.^53–55^ The preceding film's XPS analysis notably demonstrates the decrease in defects like oxygen vacancies, implying that the substrate's annealing pretreatment contributes to diminishing trap centers within the material. This, in turn, extends the duration of *τ* increase in the device.

**Fig. 5:**
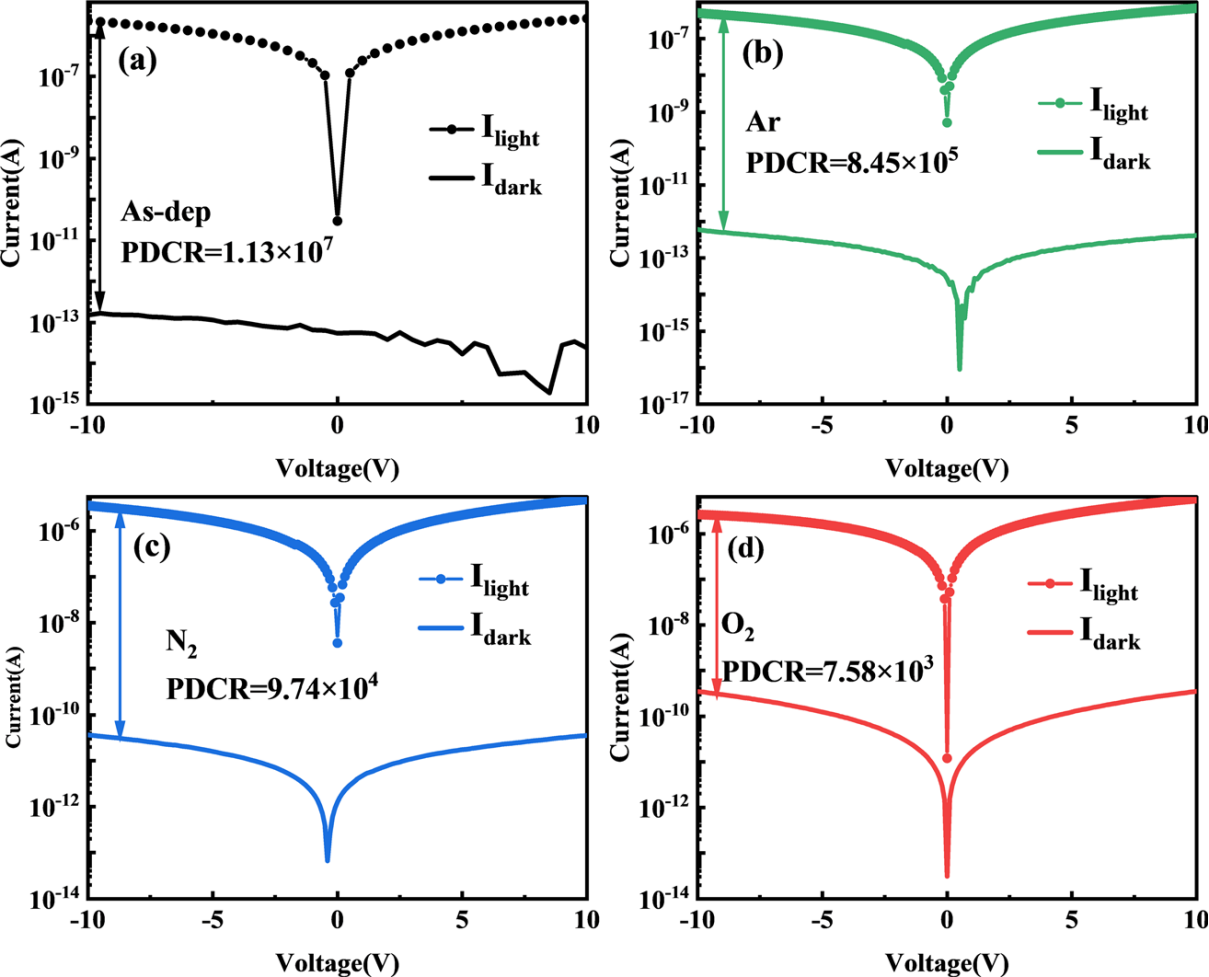
*I*–*V* characteristic curves of Ga_2_O_3_ detectors annealed under various atmospheric conditions in both dark and 254 nm illuminated states: (a) unannealed, (b) Ar-annealed, (c) N_2_-annealed, (d) O_2_-annealed.

Fig. 5 depicts the *I*–*V* characteristic curves of a gallium oxide solar-blind UV detector under a 10 V bias, while subject to varying substrate annealing conditions and exposed to both 254 nm UV light and complete darkness. The figure reveals that following the annealing pretreatment, the device experiences an increase in dark current alongside a reduction in photocurrent. To comprehend this phenomenon, an analysis of the influence of annealing on the *I*–*V* characteristics of the device was performed. It was determined that annealing treatment contributes to the reduction of oxygen vacancy concentration within the amorphous gallium oxide film. This decrease in vacancy concentration subsequently leads to diminished intrinsic carrier concentration within the film, thereby resulting in the observed reduction in the device's dark current.^35,56^

According to previous reports, oxygen vacancies in the absorption layer can trap surplus photogenerated carriers, thereby reducing electron–hole recombination and yielding higher photoconductive gain ^57,58^. Hence, the change in *I*–*V* characteristics of α-Ga_2_O_3_ photodetectors post oxygen annealing primarily stems from the decreased oxygen vacancy concentration. This perspective is also supported by the PDCR analysis. In the scenario of amorphous Ga_2_O_3_, when the device is exposed to ultraviolet light, it induces the photogenerated carriers within the α-Ga_2_O_3_ detector. These carriers then migrate towards the electrode under the influence of the applied electric field. As these photogenerated holes drift towards the Au/α-Ga_2_O_3_ interface, they are captured by the film's traps, resulting in substantial internal gain.^35,59^

The XPS analysis has previously confirmed that the unannealed films exhibit the highest oxygen vacancy content, while films annealed in an O_2_ atmosphere show the lowest content. Consequently, a reduction in the film's oxygen vacancy concentration leads to a proportional decline in the device's internal gain, subsequently influencing the device's performance.

**Fig. 6:**
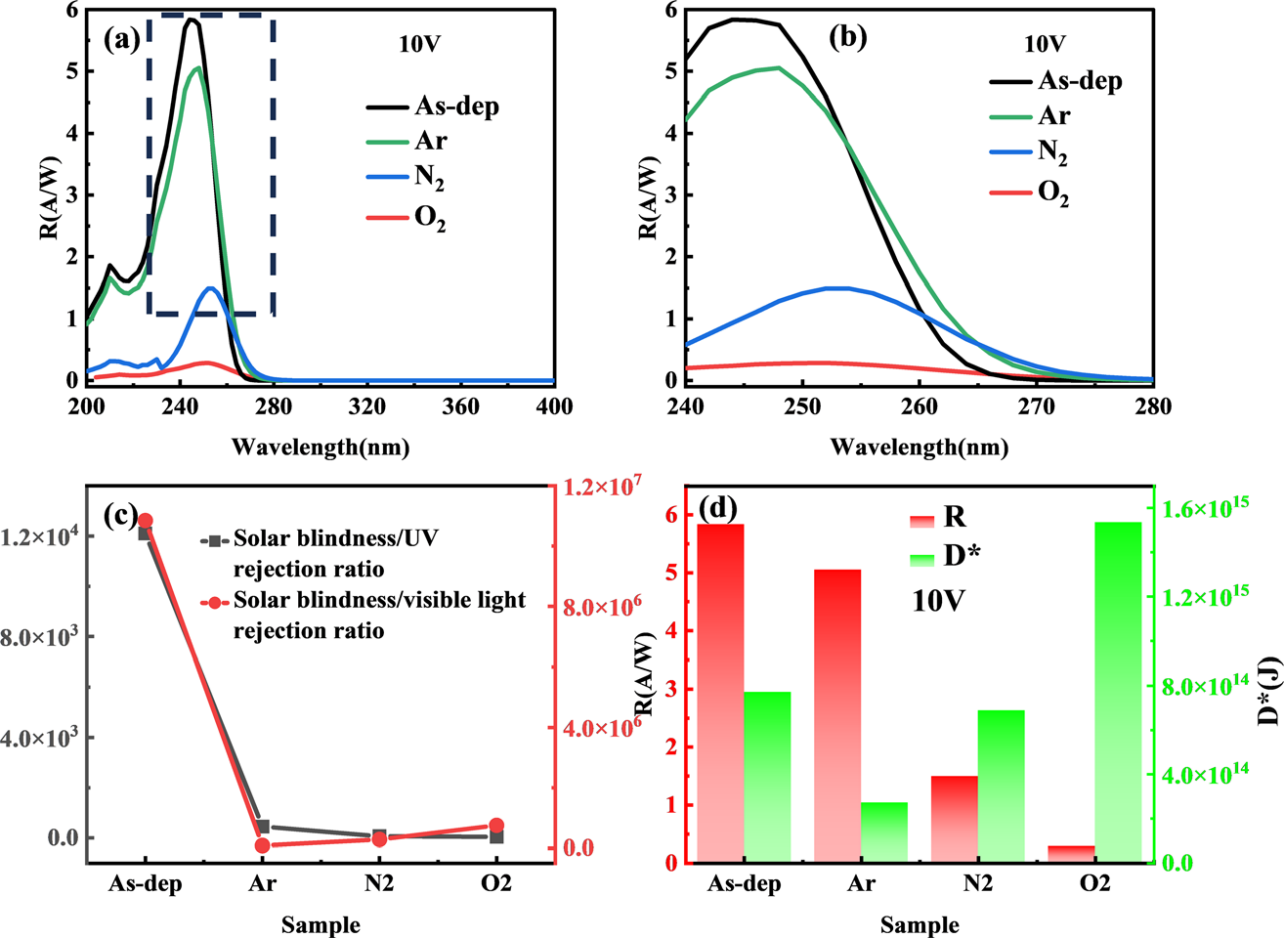
(a) PL spectra of Ga_2_O_3_ films under different annealing conditions. (b) Magnified PL spectral image within the 240–280 nm wavelength range. (c) Rejection ratio for each sample. (d) Calculation results for *R* and D* for each sample.

As a material for solar-blind UV detection, the UV spectral response characteristic curve also represents essential test data for evaluating the UV photosensitivity of gallium oxide. Fig. 6 illustrates the spectral response characteristic curve of each device within the 200–400 nm range. It is evident that the primary response band of each device is situated around 254 nm. Additionally, there is minimal to no response within the non-solar-blind region beyond 280 nm. This observation underscores the gallium oxide thin film's exceptional capability for solar-blind UV detection.

The response of the amorphous Ga_2_O_3_ detector decreased from 5.84 A W^−1^ (unannealed) to 0.29 A W^−1^ (O_2_ annealed) after annealing in different atmospheres. This decrease can be attributed to annealing's role in reducing the number of oxygen vacancies present in the absorption layer. These vacancies have the potential to trap excessive photogenerated carriers, thereby leading to a reduction in detector gain. Consequently, the device's sensitivity to visible light is significantly diminished. The solar blindness/UV rejection ratio (*R*_254nm_/*R*_280nm_, defined as the ratio of the response at 250 nm and 400 nm) and the solar blindness/visibility ratio (*R*_254nm_/*R*_400nm_, defined as the response at 250 nm and 400 nm) of the α-Ga_2_O_3_ photodetector both undergo a notable decline following annealing.

Fig. 6(d) shows the influence of distinct substrate annealing conditions on the responsiveness and detectivity of the gallium oxide solar-blind UV detector, operating under a calculated voltage of 10 V. The impact of different substrate annealing conditions on the device's responsiveness and its underlying principle closely mirrors that observed in the photocurrent analysis. Nevertheless, variations become evident in terms of normalized detectivity. Notably, solar-blind UV detectors fabricated within Ar and N_2_ atmospheres exhibit diminished detectivity when contrasted with their unannealed counterparts. In contrast, the normalized detectivity of UV detectors annealed within oxygen-rich atmospheres surpasses that of their unannealed counterparts.

The main theoretical reason is that, while the enhancement effect of mobility on the device's light and dark current remains the same, the reduction of non-intrinsic excitation resulting from improved crystal quality and diminished defects exerts an inhibitory influence on photocurrent enhancement. This impact significantly hampers device responsiveness. However, simultaneously, the increase in noise equivalent power due to elevated dark current significantly affects the device's normalized detection rate. Similarly, only an appropriate annealing atmosphere (such as the oxygen annealing atmosphere discussed in this paper) can yield a substantial enough increase in material carrier mobility to counterbalance the aforementioned inhibitory effect, consequently leading to a noteworthy enhancement in the device's normalized detectivity (Table 1).

**Table 1:** Detailed comparison between the photoresponse properties of Ga_2_O_3_ SBPD irradiated at 254 nm

	*τ*_decay_ (s) (20 V)	*I*_dark_ (A)	PDCR	*R* (A W^−1^)	D* (J)
As-dep	5.21	1.97 × 10^−13^	1.13 × 10^7^	5.836	7.72 × 10^14^
Ar	2.29	4.62 × 10^−13^	8.45 × 10^5^	5.052	2.67 × 10^14^
N_2_	0.68	2.95 × 10^−11^	9.74 × 10^4^	1.493	6.91 × 10^14^
O_2_	0.26	2.69 × 10^−10^	7.58 × 10^3^	0.287	1.57 × 10^15^

**Fig. 7:**
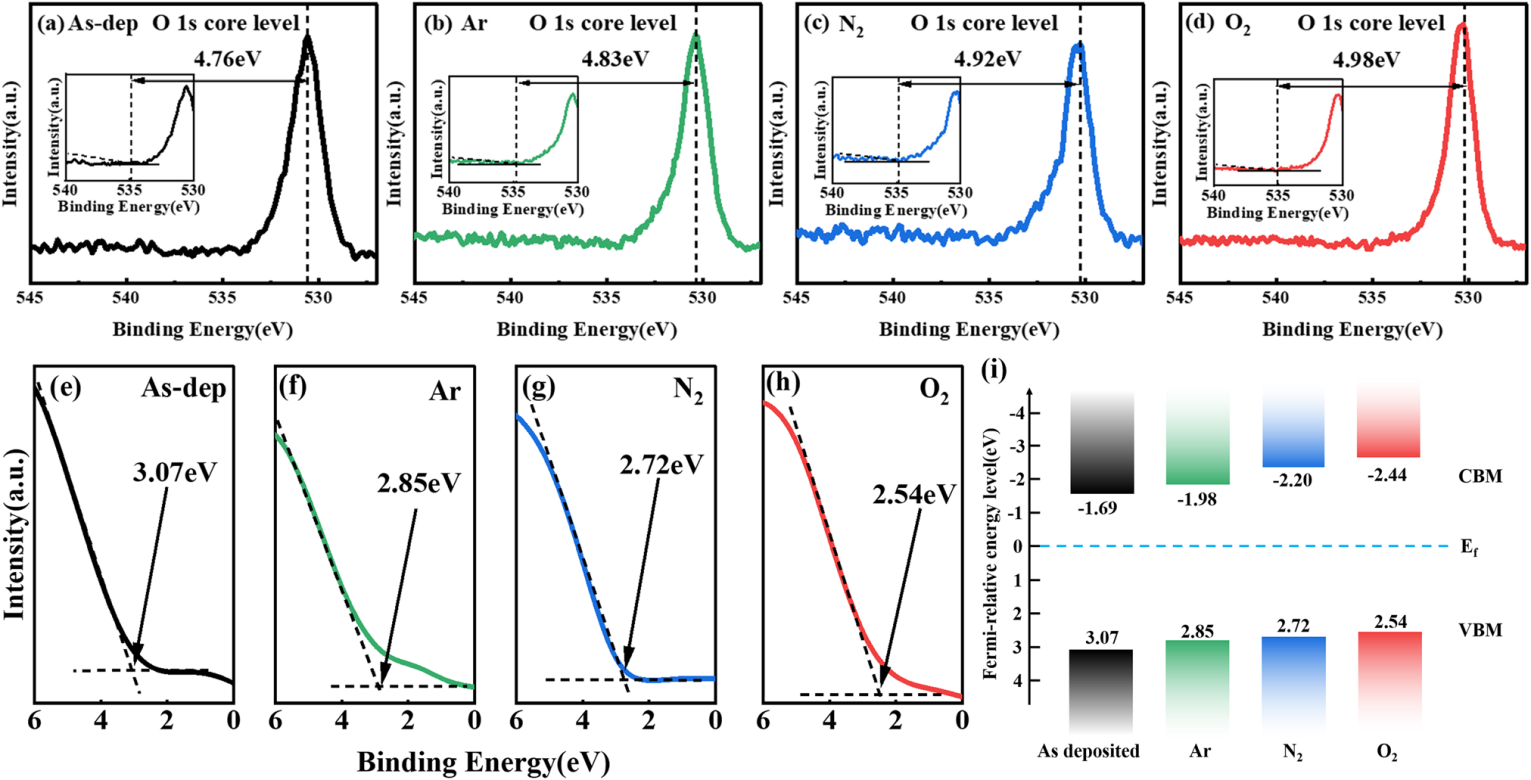
O 1s peak obtained from Ga_2_O_3_ film to determine the bandgap. The inset shows the corresponding loss structure: (a) unannealed, (b) Ar-annealed, (c) N_2_-annealed, (d) O_2_-annealed. Valence band spectra of Ga_2_O_3_ samples under distinct annealing atmospheres: (e) unannealed, (f) Ar-annealed, (g) N_2_-annealed, (h) O_2_-annealed. (i) Band shift diagram.

The influence mechanism of annealing on photoelectric detection is analyzed from the energy band perspective. Fig. 7(a)–(d) reveals the band gap width of each sample, calculated based on the XPS results of O 1s sub-peak testing. Fig. 7(e)–(h) presents the valence band spectra for each sample, forming the basis for the band shift diagram shown in Fig. 7(i). It is evident from the figure that the band gap of the amorphous gallium oxide film is 4.76 eV, whereas for the O_2_-annealed gallium oxide film, it is 4.98 eV. This implies a slightly smaller band gap width for amorphous Ga_2_O_3_ compared to annealed gallium oxide. Due to the long-term periodic destruction of the atomic arrangement and the disorderly fluctuation of the lattice potential energy, the conduction band and the valence band extend to the forbidden band to form the tailing of bands, which leads to the small band gap width. XRD results show that the crystallinity of the film increases with the increase of annealing temperature, resulting in a gradual decrease in the number of unsaturated defects. The reduction of unsaturated defects leads to a decrease in the density of defect states in the band gap, thereby reducing the tailing of bands and thereby increasing the band gap.^60,61^ Therefore, the increase of the band gap can be attributed to the decrease of the defect state density.

Furthermore, within the amorphous structure, these impurity levels exhibit higher density, consequently extending the upper valence band into the band gap region. For example, the unannealed Ga_2_O_3_ exhibits a valence band top at 1.84 eV, whereas annealing Ga_2_O_3_ in an O_2_ atmosphere shifts the valence band top further from the Fermi level to 2.47 eV. Because the top of the unannealed valence band does not deviate from the Fermi level, the band tail effect is stronger, so the band gap of the unannealed Ga_2_O_3_ film is smaller than that of the annealed Ga_2_O_3_ film. A more pronounced tail effect is likely to induce increased intrinsic excitation effects within the amorphous gallium oxide film under solar-blind ultraviolet excitation. This, in turn, leads to noteworthy alterations in solar-blind ultraviolet photosensitivity, further enhancing photocurrent, device responsiveness, and detection rate. These improvements far exceed those achieved by β-Ga_2_O_3_-based light detectors, aligning consistently with prior *R* and PDCR test outcomes.

## 4. Conclusion

In this work, we prepared amorphous Ga_2_O_3_ through ALD growth and annealed it under various atmospheres. Subsequently, we fabricated MSM SBPD. The Ga_2_O_3_-based SBPD without annealing displayed a high PDCR of 1.13 × 10^7^ and a D* of 1.32 × 10^4^ Jones. Ga_2_O_3_ films annealed in an O_2_ atmosphere exhibited a lower oxygen vacancy level (6.54%) and a faster decay time (0.17 s). Furthermore, compared to the unannealed Ga_2_O_3_ films, those annealed in an O_2_ atmosphere showed a shift of the valence band away from the Fermi level and an increased band gap. These changes directly led to reduced photocurrent and responsivity. Our results comprehensively assess the impact of different atmospheric annealing approaches on amorphous Ga_2_O_3_. The performance of the films annealed in Ar atmosphere has no obvious improvement compared with that of the unannealed films. The properties of the films annealed in N_2_ atmosphere are improved, but the effect is not as good as that in O_2_ atmosphere because new oxygen vacancies are created due to the entry of N atoms into the films. These Ga_2_O_3_ SBPDs offer a potential avenue for developing the high light response properties required for future solar-blind detection techniques.

## Author contributions

Wen-Jie Chen: data curation, formal analysis, writing – original draft. Hong-Ping Ma: methodology, supervision, funding acquisition, writing – review & editing. Lin Gu: validation, investigation. Yi Shen: investigation. Ruo-Yun Yang: data curation. Xi-Yuan Cao: investigation. Mingyang Yang: investigation. Qing-Chun Zhang: project administration, resources.

## Conflicts of interest

The authors declare that they have no known competing financial interests or personal relationships that could have appeared to influence the work reported in this paper.

## Supplementary Material

## References

[cit1] Monroy E., Omnes F., Calle F. (2003). Wide-bandgap semiconductor ultraviolet photodetectors. Semicond. Sci. Technol..

[cit2] Omnes F., Monroy E., Munoz E., Reverchon J.-L. (2007). Wide bandgap UV photodetectors: A short review of devices and applications. Proc. SPIE.

[cit3] Morkoç H., Strite S., Gao G. B., Lin M. E., Sverdlov B., Burns M. (1994). Large-band-gap SiC, III-V nitride, and II-VI ZnSe-based semiconductor device technologies. J. Appl. Phys..

[cit4] Sang L., Liao M., Sumiya M. (2013). A Comprehensive Review of Semiconductor Ultraviolet Photodetectors: From Thin Film to One-Dimensional Nanostructures. Sensors.

[cit5] Assefa S., Xia F., Vlasov Y. A. (2010). Reinventing germanium avalanche photodetector for nanophotonic on-chip optical interconnects. Nature.

[cit6] Chen H., Liu K., Hu L., Al-Ghamdi A., Fang A. X. (2015). New concept ultraviolet photodetectors. Mater. Today.

[cit7] Gao Y., Cansizoglu H., Polat K. G., Ghandiparsi S., Kaya A., Mamtaz H. H., Mayet A. S., Wang Y., Zhang X., Yamada T., Devine E. P., Elrefaie A. F., Wang S.-Y., Islam M. S. (2017). Photon-trapping microstructures enable high-speed high-efficiency silicon photodiodes. Nat. Photonics.

[cit8] Chen X., Liu K., Zhang Z., Wang C., Li B., Zhao H., Zhao D., Shen D. (2016). Self-Powered Solar-Blind Photodetector with Fast Response Based on Au/β-Ga_2_O_3_ Nanowires Array Film Schottky Junction. ACS Appl. Mater. Interfaces.

[cit9] Orita M., Ohta H., Hirano M., Hosono H. (2000). Deep-ultraviolet transparent conductive β-Ga_2_O_3_ thin films. Appl. Phys. Lett..

[cit10] Higashiwaki M., Sasaki K., Kuramata A., Masui T., Yamakoshi S. (2012). Gallium oxide (Ga_2_O_3_) metal-semiconductor field-effect transistors on single-crystal β-Ga_2_O_3_ (010) substrates. Appl. Phys. Lett..

[cit11] Cahn R. W. (1985). Eumorphous amorphs. Nature.

[cit12] Spear W. E., le Comber P. G. (1975). Substitutional doping of amorphous silicon. Solid State Commun..

[cit13] Qian L. X., Wu Z. H., Zhang Y. Y., Lai P. T., Liu X. Z., Li Y. R. (2017). Ultrahigh-Responsivity, Rapid-Recovery, Solar-Blind Photodetector Based on Highly Nonstoichiometric Amorphous Gallium Oxide. ACS Photonics.

[cit14] Guo P., Xiong J., Zhao X., Sheng T., Yue C., Tao B., Liu X. (2014). Growth characteristics and device properties of MOD derived β-Ga_2_O_3_ films. J. Mater. Sci.: Mater. Electron..

[cit15] Zhang Y., Mei Z., Cui S., Liang H., Liu Y., Du X. (2016). Flexible Transparent Field-Effect Diodes Fabricated at Low-Temperature with All-Oxide Materials. Adv. Electron. Mater..

[cit16] Rafique S., Han L., Zhao H. (2017). Thermal annealing effect on β-Ga_2_O_3_ thin film solar blind photodetector heteroepitaxially
grown on sapphire substrate. Phys. Status Solidi A.

[cit17] LEE M., YANG M., LEE H.-Y. (2020). *et al.*, The growth of HVPE α-Ga_2_O_3_ crystals and its solar-blind UV photodetector applications. Mater. Sci. Semicond. Process..

[cit18] Wang C., Li S.-W., Fan W.-H., Zhang Y.-C., Zhang X.-Y., Guo R.-R., Lin H.-J., Lien S.-Y., Zhu W.-Z. (2021). Structural, optical and morphological evolution of Ga_2_O_3_/Al_2_O_3_ (0001) films grown at various temperatures by pulsed laser deposition. Ceram. Int..

[cit19] Ji X., Yin X., Yuan Y., Yan S., Li X., Ding Z., Zhou X., Zhang J., Xin Q., Song A. (2023). Amorphous Ga_2_O_3_ Schottky photodiodes with high-responsivity and photo-to-dark current ratio. J. Alloys Compd..

[cit20] Chen X., Xu Y., Zhou D., Yang S., Ren F., Lu H., Tang K., Gu S., Zhang R., Zheng Y., Ye J. (2017). Solar-Blind Photodetector with High Avalanche Gains and Bias-Tunable Detecting Functionality Based on Metastable Phase α-Ga_2_O_3_/ZnO Isotype Heterostructures. ACS Appl. Mater. Interfaces.

[cit21] Oshima T., Okuno T., Fujita S. (2007). Ga_2_O_3_ Thin Film Growth on c-Plane Sapphire Substrates by Molecular Beam Epitaxy for Deep-Ultraviolet Photodetectors. Jpn. J. Appl. Phys..

[cit22] Yu F. P., Ou S. L., Wuu D. S. (2015). Pulsed laser deposition of gallium oxide films for high performance solar-blind photodetectors. Opt. Mater. Express.

[cit23] Zhang J., Li B., Xia C., Pei G., Deng Q., Yang Z., Xu W., Shi H., Wu F., Wu Y., Xu J. (2006). Growth and spectral characterization of β-Ga_2_O_3_ single crystals. J. Phys. Chem. Solids.

[cit24] Wang Y., Pu T., Li X., Li L., Ao J. P. (2021). Application of p-type NiO deposited by magnetron reactive sputtering on GaN vertical diodes. Mater. Sci. Semicond. Process..

[cit25] Rawal S. K., Chawla A. K., Chawla V., Jayaganthan R., Chandra R. (2010). Effect of ambient gas on structural and optical properties of titanium oxynitride films. Appl. Surf. Sci..

[cit26] Liang Y. (2013). Preparation of Nanometer Sized Cuprous Oxide and its Photocatalytic Performance over Four Nitrophenol. Appl. Mech. Mater..

[cit27] Dong L., Jia R., Xin B., Peng B., Zhang Y. (2017). Effects of oxygen vacancies on the structural and optical properties of β-Ga_2_O_3_. Sci. Rep..

[cit28] Wang F.-H., Chen K.-N., Hsu C.-M., Liu M.-C., Yang C.-F. (2016). Investigation of the Structural, Electrical, and Optical Properties of the Nano-Scale GZO Thin Films on Glass and Flexible Polyimide Substrates. Nanomaterials.

[cit29] Ma H. P., Lu H. L., Yang J. H., Li X. X., Wang T., Huang W., Yuan G.-J., Komarov F., Zhang D. (2018). Measurements of Microstructural, Chemical, Optical, and Electrical Properties of Silicon-Oxygen-Nitrogen Films Prepared by Plasma-Enhanced Atomic Layer Deposition. Nanomaterials.

[cit30] Ma H. P., Lu H. L., Wang T., Yang J. G., Li X., Chen J. X., Tao J. J., Zhu J. T., Guo Q., Zhang D. W. (2018). Precise control of the microstructural, optical, and electrical properties of ultrathin Ga_2_O_3_ film through nanomixing with few atom-thick SiO_2_ interlayer via plasma enhanced atomic layer deposition. J. Mater. Chem. C.

[cit31] Hedei P. H. M. A., Hassan Z., Quah H. J. (2021). Effects of post-deposition annealing temperature in nitrogen/oxygen/nitrogen ambient on polycrystalline gallium oxide films. Appl. Surf. Sci..

[cit32] Chen Y. C., Chen D., Zeng G., Li X., Li Y. C., Zhao X. F., Chen N., Wang T., Zhang D. W., Lu H. L. (2023). High Performance Solar-Blind Photodetectors Based on Plasma-Enhanced Atomic Layer Deposition of Thin Ga_2_O_3_ Films Annealed Under Different Atmosphere. J. Alloys Compd..

[cit33] Ma Y. B., Duan P., Deng J. X., Wang J. Y., Cui M., Kong L. (2017). Effect of post-annealing on β-gallium oxide thin films deposited by RF magnetron sputtering. Vacuum.

[cit34] Zhang H., Deng J., Kong L., Pan Z., Bai Z., Wang J. (2019). Effect of annealing atmosphere on the structural and optical properties of the Nb-doped β-Ga_2_O_3_ films. Micro Nano Lett..

[cit35] Dong L., Jia R., Xin B., Peng B., Zhang Y. (2017). Effects of oxygen vacancies on the structural and optical properties of β-Ga_2_O_3_. Sci. Rep..

[cit36] Razeghi M., Rogalski A. (1996). Semiconductor ultraviolet detectors. J. Appl. Phys..

[cit37] Feng W., Wang X., Zhang J., Wang L., Zheng W., Hu P., Cao W., Yang B. (2014). Synthesis of two-dimensional β-Ga_2_O_3_ nanosheets for high-performance solar blind photodetectors. J. Mater. Chem. C.

[cit38] Huang C. Y., Horng R. H., Wuu D. S., Tu L. W., Kao H.-S. (2013). Thermal annealing effect on material characterizations of β-Ga_2_O_3_ epilayer grown by metal organic chemical vapor deposition. Appl. Phys. Lett..

[cit39] Tien L. C., Chen W. T., Ho C. H. (2011). Enhanced Photocatalytic Activity in β-Ga_2_O_3_ Nanobelts. J. Am. Ceram. Soc..

[cit40] Yang H., Shi R., Yu J., Liu R., Zhang R., Zhao H., Zhang L., Zheng H. (2009). Single-Crystalline β-Ga_2_O_3_ Hexagonal Nanodisks: Synthesis, Growth Mechanism, and Photocatalytic Activities. J. Phys. Chem. C.

[cit41] Shi F., Zhang S., Xue C. (2010). Influence of annealing time on microstructure of one-dimensional Ga_2_O_3_ nanorods. J. Alloys Compd..

[cit42] Hou X., Zou Y., Ding M., Qin Y., Zhang Z., Ma X., Tan P., Yu S., Zhou X., Zhao X., Xu G., Sun H., Long S. (2020). Review of polymorphous Ga_2_O_3_ materials and their solar blind photodetector applications. J. Phys. D Appl. Phys..

[cit43] Shi L., Chen K., Zhai A., Li G., Fan M., Hao Y., Zhu F., Zhang H., Cui Y. (2020). Status and outlook of metal–inorganic semiconductor–metal photodetectors. Laser Photonics Rev..

[cit44] Alaie Z., Mohammad Nejad S., Yousefi M. H. (2015). Recent advances in ultraviolet photodetectors. Mater. Sci. Semicond. Process..

[cit45] Kufer D., Konstantatos G. (2016). Photo-FETs: Phototransistors Enabled by 2D and 0D Nanomaterials. ACS Photonics.

[cit46] Yao Y., Gangireddy R., Kim J., Das K. K., Davis R. F., Porter L. M. (2017). Electrical behavior of β-Ga_2_O_3_ schottky diodes with different schottky metals. J. Vac. Sci. Technol. B.

[cit47] Oh S., Jung Y., Mastro M. A., Hite J. K., Eddy Jr C. R., Kim J. (2015). Development of solar blind photodetectors based on Si-implanted β-Ga_2_O_3_. Opt. Express.

[cit48] Guo D., Wu Z., Li P., An Y., Liu H., Guo X., Yan H., Wang G., Sun C., Li L., Tang W. (2014). Fabrication of β-Ga_2_O_3_ films and solar-blind photodetectors by laser MBE technology. Opt. Mater. Express.

[cit49] Guo D. Y., Wu Z. P., An Y. H. (2014). Oxygen vacancy tuned Ohmic-Schottky conversion for enhanced performance in β-Ga_2_O_3_ solar-blind ultraviolet photodetectors. Appl. Phys. Lett..

[cit50] Guo D., Wu Z. (2014). Fabrication of β-Ga_2_O_3_ thin films and solar-blind photodetectors by laser MBE technology. Opt. Mater. Express.

[cit51] Weng W. Y., Hsueh T. J. (2011). a β-Ga_2_O_3_ solar-blind photodetector prepared by furnace oxidization of GaN thin film. IEEE Sensor. J..

[cit52] Sigaev V. N., Golubev N. V. (2014). Light-emitting Ga-oxide nanocrystals in glass: a new paradigm for low-cost and robust UV-to-visible solar-blind converters and UV emitterst. Nanoscale.

[cit53] Visoly-Fisher I., Cohen S. R. (2006). Understanding the beneficial role of grain boundaries in polycrystalline solar cells from single-grain-boundary scanning probe microscopy. Adv. Funct. Mater..

[cit54] Hetzera M. J., Strzhemechny Y. M. (2005). Direct observation of copper depletion and potential changes at copper indium gallium diselenide grain boundaries. Appl. Phys. Lett..

[cit55] Zych E., Brecher C. (1997). Luminescence properties of Ce-activated YAG optical ceramic scintillator materials. J. Lumin..

[cit56] Nakamur I., Negishi N. (2000). Role of oxygen vacancy in the plasma-treated TiO photocatalyst with visible light activity for NO removal. J. Mol. Catal. Chem..

[cit57] Ye Z., Wong M. (2012). Characteristics of plasma-fluorinated Zinc Oxide thin-film transistors. IEEE Electron Device Lett..

[cit58] Jung H., Kim D., Kim H. (2014). The electrical properties of low pressure chemical vapor deposition Ga doped ZnO thin films depending on chemical bonding configuration. Appl. Surf. Sci..

[cit59] Skuja L. (1998). Optically active oxygen-deficiency-related centers in amorphous silicon dioxide. J. Non-Cryst. Solids.

[cit60] Khan S. A., Zulfequar M., Husain M. (2002). Effects of annealing on crystallization process in amorphous Ge_5_Se_95_−xTex thin films. Phys. B.

[cit61] Shi Q., Wang Q., Zhang D., Wang Q., Li S., Wang W., Fan Q., Zhang J. (2019). Structural, optical and photoluminescence properties of Ga_2_O_3_ thin films deposited by vacuum thermal evaporation. J. Lumin..

